# Common mechanisms for type 2 diabetes and psychosis: Findings from a prospective birth cohort

**DOI:** 10.1016/j.schres.2020.08.006

**Published:** 2020-09

**Authors:** Benjamin I. Perry, Hannah J. Jones, Tom G. Richardson, Stan Zammit, Nicholas J. Wareham, Glyn Lewis, Peter B. Jones, Golam M. Khandaker

**Affiliations:** a Department of Psychiatry, University of Cambridge School of Clinical Medicine, Cambridge, England, UK; b Cambridgeshire and Peterborough NHS Foundation Trust, Cambridge, England, UK; c Centre for Academic Mental Health, Population Health Sciences, Bristol Medical School, University of Bristol, Bristol, England, UK; d NIHR Biomedical Research Centre, University Hospitals Bristol NHS Foundation Trust and University of Bristol, Bristol, UK; e MRC Integrative Epidemiology Unit, Population Health Sciences, Bristol Medical School, University of Bristol, Bristol, England, UK; f MRC Centre for Neuropsychiatric Genetics and Genomics, Cardiff University, Cardiff, Wales, UK; g MRC Epidemiology Unit, University of Cambridge School of Clinical Medicine, Cambridge, England, UK; h Division of Psychiatry, University College London, London, England, UK

**Keywords:** Psychosis, Schizophrenia, Diabetes mellitus, Polygenic risk, ALSPAC

## Abstract

**Background:**

Psychosis and type 2 diabetes mellitus (T2DM) are commonly comorbid and may share pathophysiologic mechanisms. To investigate shared genetic variation and inflammation as potential common mechanisms, we tested: (i) associations between genetic predisposition for T2DM and psychotic experiences and psychotic disorder in young adults; (ii) the association between genetic predisposition for schizophrenia and insulin resistance (IR), a precursor of T2DM; and (iii) whether these associations are mediated by childhood inflammation.

**Methods:**

Psychotic experiences (PEs), psychotic disorder and IR were assessed at age 18. Polygenic risk scores (PRS) for T2DM and schizophrenia were derived based on large genome-wide association studies. Associations between PRS and psychotic/IR outcomes were assessed using regression analysis based on 3768 ALSPAC birth cohort participants with complete data. Inflammatory markers C-reactive protein (CRP) and interleukin 6 (IL-6) measured at age 9 were used in regression and mediation analyses.

**Results:**

Genetic predisposition for T2DM was associated with PEs (adjusted OR = 1.21; 95% CI, 1.01–1.45) and psychotic disorder (adjusted OR = 1.51; 95% CI, 1.04–2.03) at age 18 in a linear dose-response fashion. Genetic predisposition for schizophrenia was weakly associated with IR (adjusted OR = 1.10; 95% C·I, 0.99–1.22) at age 18. The association between genetic risk for T2DM and PEs was partly mediated by childhood CRP (*p* = .040).

**Conclusions:**

Comorbidity between psychosis and T2DM may be partly underpinned by shared genes and inflammation. A summation of minor genetic variation representing lifetime risk for T2DM at conception may predispose individuals to psychosis in adulthood by influencing physiologic changes, such as low-grade inflammation, detectable as early as childhood.

## 1 Introduction

Reduced life-expectancy in schizophrenia is largely attributable to physical comorbidity including cardiometabolic disorders, which are up to 30% more prevalent in people with schizophrenia than in the general population (Holt et al., 2004) (Lappin et al., 2018). Compared with controls, markers of abnormal glucose-insulin homeostasis are two to three times higher in young people with psychotic experiences (PEs) (Perry et al., 2018), and in medication-naive first-episode psychosis (FEP) (Perry et al., 2016; Pillinger et al., 2017) after controlling for anthropometric and sociodemographic factors. This suggests that increased T2DM in patients with psychosis may not be fully explained by common lifestyle factors or side-effects of antipsychotic drugs, though may be exacerbated by them (Rajkumar et al., 2017).

One contributor to comorbidity between cardiometabolic disorders and schizophrenia could be shared genetic susceptibility (Lin and Shuldiner, 2010). Risk of insulin resistance (IR) (Chouinard et al., 2019) and impaired glucose tolerance (Ferentinos and Dikeos, 2012), two key precursors of T2DM, are higher in unaffected relatives of patients with psychosis compared with controls. People with comorbid schizophrenia and T2DM have a higher genetic predisposition for both disorders compared to controls (Hackinger et al., 2018), and an association between genetic predisposition for schizophrenia and IR has been reported in a clinical sample (Tomasik et al., 2019). Conversely, a relatively small study found no evidence of an association between genetic risk for T2DM and psychosis (Padmanabhan et al., 2016), and previous research using linkage-disequilibrium (LD) score regression found limited evidence for a genetic correlation between schizophrenia and T2DM (Bulik-Sullivan et al., 2015). However, a key feature of existing studies is that they are based on adult cases of established schizophrenia or T2DM or rely on blood measurements taken in adulthood, so confounding by cumulative effects of lifestyle and other factors is possible (Reinikainen et al., 2015). Population-based prospective studies have identified early markers of disease risk associated with T2DM and schizophrenia. For instance, PEs in adolescence or young adulthood are associated with risk of schizophrenia in adulthood (Poulton et al., 2000; Zammit et al., 2013), and IR is a precursor of T2DM (Martin et al., 1992). To our knowledge, no studies have examined whether genetic predispositions for T2DM or schizophrenia are associated with, respectively, PEs or IR, in young adulthood. Demonstrating such associations with early markers of illness in young adults with lessened effects of cumulative lifestyle confounding would be consistent with the idea that shared genetic variation is a common mechanism for comorbid T2DM and schizophrenia.

Although existing studies provide some evidence for a shared genetic basis for T2DM and schizophrenia, underlying pathophysiologic mechanisms remain unclear. Low-grade inflammation may be one such mechanism, which has been reported to be associated with IR (Festa et al., 2000), T2DM (Pradhan et al., 2001) and psychosis (Upthegrove et al., 2014). Population-based longitudinal studies report that higher levels of circulating inflammatory markers at baseline are associated with risks of psychosis and abnormal glucose-insulin homeostasis subsequently at follow-up (Khandaker et al., 2014; Perry et al., 2018). Mendelian randomisation (MR) studies have reported associations of genetic variants regulating inflammatory biomarkers such as interleukin-6 (IL-6) with schizophrenia (Hartwig et al., 2017), suggesting that inflammation may be associated with schizophrenia beyond any effects of confounding. Inflammation has also been implicated in the pathogenesis of IR and T2DM (Pradhan et al., 2001).

We examined whether shared genetic variation and inflammation could be common mechanisms for T2DM and psychosis using prospective, population-based data from the ALSPAC birth cohort. We tested whether: (i) genetic predisposition for T2DM is associated with risk of PEs and psychotic disorder at age 18; (ii) genetic predisposition for schizophrenia is associated with IR at age 18; (iii) whether these associations are mediated by CRP or IL-6 levels measured in childhood at age 9.

## 2 Methods

### 2.1 Description of cohort and sample selection

The ALSPAC birth cohort (Boyd et al., 2013; Fraser et al., 2013) comprises 14,062 live births from mothers residing in former County Avon in Southwest England, with expected dates of delivery between April 1991 and December 1992 (http://www.bristol.ac.uk/alspac/researchers/our-data/). The study received ethics approval from the ALSPAC Ethics and Law Committee and local research ethics committees. All participants provided written or implied informed consent. In total, 7977 participants had genotyping data, 3768 participants had data on both genotyping and psychosis outcomes, and 2344 participants had data on genotyping and IR (Supplementary Fig. 1). Our analysis was conducted on participants without missing data for the covariates or outcomes of interest.

### 2.2 Assessment of psychotic outcomes at age 18

#### 2.2.1 Psychotic experiences (PEs)

PEs were identified through the face-to-face, semi-structured Psychosis-Like Symptom Interview (PLIKSi) conducted by trained psychology graduates. The PLIKSi comprised of an introductory set of questions on unusual experiences, and then 12 ‘core’ questions eliciting key symptoms covering the three main domains of positive psychotic symptoms: hallucinations (visual and auditory); delusions (delusions of being spied on, persecution, thoughts being read, reference, control, grandiose ability and other unspecified delusions); and symptoms of thought interference (thought broadcasting, insertion and withdrawal). For these 12 core items, 7 stem questions were derived from the Diagnostic Interview Schedule for Children–IV (DISC–IV) and 5 stems from section 17–19 of the Schedules for Clinical Assessment in Neuropsychiatry version 2.0 (SCAN 2.0). After cross-questioning, interviewers rated PEs as not present, suspected, or definitely present. Interviewers rated down (i.e. suspected rather than definite, or none rather than suspected) if unsure. For suspected or definite PEs, interviewers also recorded the frequency; effects on social/educational/ occupational function; help seeking; and attributions including fever, hypnopompic/hypnogogic state, or illicit drugs. For interrater reliability, the interviewers recorded audio interviews at three time points, approximately 6 months apart, across the clinic duration (75 interviews in total). The average kappa value of PEs was 0.83, with no evidence of differences across time. Test-retest reliability was assessed using 162 individuals reinterviewed after approximately 47 days (kappa = 0.76, SE = 0.078), 46 of whom were reinterviewed by the same interviewer (kappa = 0.86, SE = 0.136). Our primary outcome was presence of *definite* PEs, referring to at least one definite PE since age 12; the comparator group was suspected/no PEs. Our outcome is reflective of 6-year period prevalence of definite PEs. From the total number of participants with definite PEs at 18y (230, 4.9%), 80 participants (45.3%) had suffered definite PEs at least once in the month preceding assessment. From the total sample of participants reporting definite PEs, 146 participants (63.5%) reported auditory hallucinations, 63 participants (28.2%) reported any delusion, and 22 participants (9.9%) reported thought disturbance. See Supplementary Table 1 for full frequency data, Supplementary Table 2 for information on timing of onset of PEs, and the main reporting study for further information (Zammit et al., 2013).

#### 2.2.2 Psychotic disorder

Psychotic disorder was defined (Zammit et al., 2013) as the presence of PEs when symptoms were not attributable to fever/sleep/drugs, had occurred at least once per month over the previous 6 months, and caused significant distress resulting in either help-seeking from a professional source (general practitioner, counsellor, mental health team), or significantly disrupted social/occupational function. From the total ALSPAC sample who underwent the PLIKSi, 46 participants (1.0%) met criteria for psychotic disorder. We included psychotic disorder as a secondary outcome due to its lower prevalence in the study sample.

### 2.3 Assessment for a T2DM-risk outcome at age 18

#### 2.3.1 Insulin resistance

IR was calculated as a binary variable based on fasting plasma glucose and insulin levels at age 18, using the well-validated homeostasis model assessment (HOMA) method (Matthews et al., 1985). There is no consensus-agreed cut-off for clinical IR in the literature since levels can vary between populations (Wallace et al., 2004). Therefore, we used the 75th centiles of the study population to define IR. The 75th centile cut-off has been used in previous research (Cediel et al., 2016; Geloneze et al., 2006; Hedblad et al., 2000; Marques-Vidal et al., 2002). The 75th centile in our study population was 2.15.

### 2.4 Assessment for polygenic risk scores for T2DM and schizophrenia

From the ALSPAC cohort, 8812 participants were genotyped using the Illumina HumanHap550 quad genome-wide SNP genotyping platform by *23andMe* subcontracted to the Wellcome Trust Sanger Institute, Cambridge, UK and the Laboratory Corporation of America, Burlington, NC, USA. Following quality control assessment and imputation, and restricting to 1 young person per family, genetic data was available for 7977 ALSPAC individuals. See Supplementary Methods for further information.

Polygenic risk scores (PRS) for schizophrenia and T2DM were constructed for all 7977 participants with genotype data, using training sets based on the second Psychiatric Genomics Consortium (PGC) Schizophrenia GWAS (Schizophrenia Working Group of the Psychiatric Genomics Consortium, 2014) and a large T2DM GWAS (Mahajan et al., 2014), respectively. Both GWAS analyses adjusted for principal components to reduce the impact of population stratification (Price et al., 2006). PRS were calculated using the PLINK (v1.9) (Chang et al., 2015; Purcell et al., 2007) ‘score’ command following the methodology described by the International Schizophrenia Consortium (ISC) (Purcell et al., 2009). Prior to construction of scores, single nucleotide polymorphisms (SNPs) were removed from the analysis if they had a minor allele frequency less than 0.01, an imputation quality less than 0.8 or if there was allelic mismatch between samples (see Supplementary methods for details). Due to the presence of strand differences between ALSPAC and the T2DM GWAS, and lack of allele frequency information in the T2DM summary statistics, palindromic SNPs were also removed prior to construction of the T2DM PRS. Because of the high linkage disequilibrium (LD) within the extended major histocompatibility complex (MHC; chromosome 6: 25-34 Mb) only a single SNP was included to represent this region. SNPs were pruned for LD using the PLINK ‘clump’ command to remove SNPs in LD (*r*^2^ > 0.25) with a more significant SNP in the training set. Windows of 500 kb were used to assess inter-SNP LD for pruning.

For the primary analysis, PRS were constructed using a list of SNPs with the optimal *p*-value thresholds to capture phenotypic variance defined by both GWAS individually (*p* ≤ 10^−5^ for T2DM (Mahajan et al., 2014) and *p* ≤ .05 for schizophrenia (Schizophrenia Working Group of the Psychiatric Genomics Consortium, 2014)). Scores were weighted by the logarithm of the odds ratio (OR) for schizophrenia or T2DM reported by the GWAS training sets, for the schizophrenia and T2DM PRS, respectively. 10 Principal components (PCs) were generated using unrelated individuals (IBS < 0.05) and independent SNPs (with long range LD regions removed) using the `-- pca` command in PLINK1.90. All PRS analyses were adjusted for the 10 PCs to reduce the risk of population stratification. Two PRS measures were calculated for T2DM; the first including all SNPs associated with T2DM, and the second after excluding a SNP located in the *FTO* gene region, which is widely understood to be associated with T2DM only through its influence on body mass index (BMI) variation (Frayling et al., 2007); the latter was used in sensitivity analysis. Additionally, since the optimal *p*-value thresholds of both PRS scores differed, we conducted sensitivity analyses to examine PRS-outcome associations using a range of *p*-value thresholds from *p* = .5 to genome-wide significance (*p* < 5 × 10^−8^).

### 2.5 Assessment of inflammatory markers at age 9

Data on two inflammatory markers at age 9 years (IL-6 and CRP) were available in ALSPAC, for 5076 and 5086 participants respectively. Blood samples were collected at non-fasting state. Please see supplementary methods for further information.

### 2.6 Assessment of potential confounders

We included sex (categorical), ethnicity (binary caucasian/non-caucasian due to the predominantly caucasian sample), social class (categorical) and BMI at age 18 years (continuous). We excluded participants with hsCRP levels >10 mg/L to minimize potential bias from recent/ongoing infection or chronic inflammatory disease.

### 2.7 Statistical analysis

We examined the distribution of PRS-T2DM and PRS-schizophrenia using the Shapiro-Wilk test for normality, and from visual inspection of Q-Q plots. The distributions were *p* > .05 and appeared normally distributed. Both PRS variables were standardized (*Z*-transformed).

#### 2.7.1 Association between PRS and outcomes at age 18

We conducted logistic regression analyses to examine the association between PRS-T2DM and risks for PEs and psychotic disorder, and PRS-schizophrenia and IR at age 18. The odds ratios (OR) and 95% confidence intervals (95% C.I.) indicate increase in risk per standard deviation (SD) increase in PRS. Regression models were adjusted for sex, ethnicity, social class, and BMI. *p*-values for adjusted regression models in our primary analysis were corrected for multiple testing per the three outcomes we included (definite PEs, psychotic disorder and IR) using the Holm-Bonferroni method (Holland and Copenhaver, 1987). We used the p.adjust() command in R (R Core Team, 2017) to perform adjustments. In results tables, we present the original unadjusted *p*-values alongside Holm-Bonferroni adjusted *p*-values. To test for linearity of associations, we included a quadratic term (PRS^2^) in the logistic regression models.

#### 2.7.2 Association between PRS scores and childhood inflammatory markers at age 9 years

We used linear regression analyses to test associations of PRS for T2DM or schizophrenia, separately, with IL-6 and CRP levels at age 9 years (Z-transformed values), before and after adjustments for potential confounders listed above.

#### 2.7.3 Mediation by childhood CRP

We performed mediation analyses to examine whether any evident associations may be mediated by childhood CRP levels. We calculated direct and indirect effects between exposure (PRS-T2DM or PRS-schizophrenia) and outcome (e.g., PEs or IR) taking into account the mediator variable (e.g., CRP). Evidence of an indirect effect is consistent with mediation. The indirect effect was bootstrapped using 5000 iterations to determine the 95% CIs. Mediation analysis was performed using the PROCESS macro V3.1 for IBM SPSS 24.0 (http://www.afhayes.com).

### 2.8 Missing data

We assessed the potential impact of missing data by comparing mean PRS score between the analytic sample and participants with missing data for psychosis and IR outcomes, using separate variance *t*-tests. We also performed logistic regression analysis to determine sociodemographic and other predictors (sex, ethnicity, BMI and social class) of missing data.

## 3 Results

### 3.1 Baseline characteristics of sample

Of the 3768 participants with data on PRS-T2DM and psychotic outcomes, 283 met the criteria for suspected/definite PEs (7.5%), 183 for definite PEs (5.1%), 29 (0.7%) for psychotic disorder at age 18 (Table 1). Of the 2344 participants with data on PRS-schizophrenia and IR, 173 met the criteria for IR at age 18 (7.3%).

**Table 1:** Baseline characteristics of sample.

Characteristic, *n* (%) unless otherwise stated	All sample	Definite PEs	Psychotic disorder	No/suspected PEs
Male sex	1846 (49)	71 (38)	7 (15)	1775 (49)
White British ethnicity	3692 (98)	179 (98)	39 (95)	3513 (98)
Social class				
I & II	1582 (42)	62 (35)	5 (16)	1456 (40)
III - non manual & manual	1616 (43)	75 (43)	15 (48)	1630 (44)
IV & V	565 (15)	38 (22)	11 (36)	583 (16)
BMI (kg/m^2^) at 18 years, mean (SD)	22.71 (3.76)	23.37 (4.49)	22.73 (4.26)	22.60 (3.71)
HOMA at 18 years, mean (SD)	0.92 (0.73)	1.03 (0.75)	1.28 (1.00)	0.92 (0.73)
Insulin resistance	251 (8)	25 (17)	7 (20)	209 (7)
Current smoking	220 (7)	22 (15)	5 (18)	188 (7)
CRP (mg/L) at 9 years, mean (SD)	0.68 (2.52)	0.72 (2.61)	0.75 (1.33)	0.67 (2.49)
PEs attributed to sleep/fever/drugs^a^	N/A	31 (0.7)	7 (0.1)	27 (0.6)
Help-seeking from professional source^a^	N/A	55 (24)	41 (51.9)	6 (3)

Information based on total ALSPAC sample.

a Recorded from Zammit et al. (2013).

### 3.2 Association between genetic predisposition for T2DM and psychotic outcomes at age 18

**Fig. 1:**
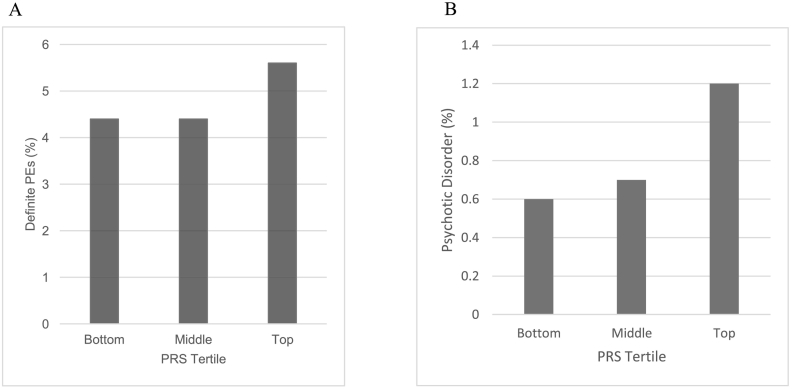
Prevalence of psychotic experiences and psychotic disorder at age 18 per tertile of PRS-T2DM.

The prevalence of psychotic outcomes at age 18 years was higher for participants in the top third of PRS-T2DM distribution compared with those in the bottom third (Fig. 1). PRS-T2DM was associated with definite PEs (adjusted OR = 1.21; 95% CI, 1.01–1.45 per SD increase in PRS-T2DM) and psychotic disorder (adjusted OR = 1.51; 95% CI, 1.04–2.05 per SD increase in PRS-T2DM) at age 18 years after controlling for sex, ethnicity, social class and BMI (Table 2). Quadratic terms for PRS-T2DM in these regression models were non-significant suggesting no evidence for departure from linearity (all *p* > .05). The results for sensitivity analyses using PRS-T2DM score excluding a SNP in the *FTO* gene region were similar (Supplementary Table 3).

**Table 2:** Odds ratios (95% CI) for outcomes at age 18 per SD increase in polygenic risk score for T2DM or schizophrenia.

Outcome/risk factor	Sample	OR (95% C.I.)		*p*-value	Corrected *p*-value^c^
		Unadjusted^a^	Adjusted for sex, ethnicity, social class and BMI^b^		
Definite PEs					
PRS-T2DM	3768	1.15 (0.99–1.34)	1.21 (1.01–1.45)	0.027	0.054

Psychotic disorder					
PRS-T2DM	3768	1.42 (1.00–1.96)	1.51 (1.04–2.05)	0.016	0.048⁎

Insulin resistance					
PRS-SCZ	2344	1.16 (1.04–1.32)	1.10 (0.99–1.22)	0.089	0.089

a Unadjusted analysis adjusted for 10 principal components only.

b Samples for adjusted analysis included 3070 participants for psychotic outcomes and 1970 participants for insulin resistance outcome.

c *p*-value corrected from adjusted analysis using Holm-Bonferroni method.

⁎ Evidence surpasses Holm-Bonferroni threshold.

### 3.3 Association between genetic predisposition for schizophrenia and IR at age 18

There was weaker evidence for an association between PRS-schizophrenia and IR at age 18 (adjusted OR = 1.10; 95% CI, 0.99–1.22 per SD increase in PRS-schizophrenia) after controlling for sex, ethnicity, social class and BMI. The quadratic term for PRS-schizophrenia was non-significant suggesting no evidence for departure from linearity (*p* > .05).

### 3.4 Associations between PRS scores and inflammatory markers at age 9

Data on both PRS scores and serum IL-6 and CRP levels were available for 2180 and 2176 participants respectively. After adjustments for sex, ethnicity, social class and BMI, PRS-T2DM was associated with CRP (β = 0.03; 95% CI, 0.01–0.08, *p* = .040), but not with IL-6 (β = 0.01; 95% CI, −0.02–0.05, *p* = .082). There was also trend level evidence for an association between PRS-schizophrenia and CRP (β = 0.05; 95% CI, −0.01–0.10, *p* = .061) but not with IL-6 (β = 0.01; 95% CI, −0.04-0.09, *p* = .670).

### 3.5 Mediating effect of childhood CRP levels on the associations of PRS scores with psychotic outcomes or IR

Based on 1955 participants with data on PRS-T2DM, CRP levels at age 9 and PEs at age 18, CRP at age 9 partially mediated the association between PRS-T2DM and definite PEs at age 18. There was evidence of an indirect effect indicative of mediation; the coefficients were 0.28; 95% CI, 0.07–0.45, *p* = .044 for direct effect; co-efficient = 0.05; 95% CI 0.02–0.12, *p* = .040 for indirect effect. Since IL-6 levels at age 9 years were not associated with PRS-T2DM, we did not perform mediation analysis using IL-6. There was no evidence for a mediating effect of CRP on the association between PRS-schizophrenia and IR at age 18; the coefficients were 0.14; 95% CI, −0.06–0.34, *p* = .756 for direct effect; co-efficient = 0.01; 95% CI, −0.01–0.03, *p* = .180 for indirect effect.

### 3.6 Results for sensitivity analysis using different *P*-value thresholds for PRS

**Fig. 2:**
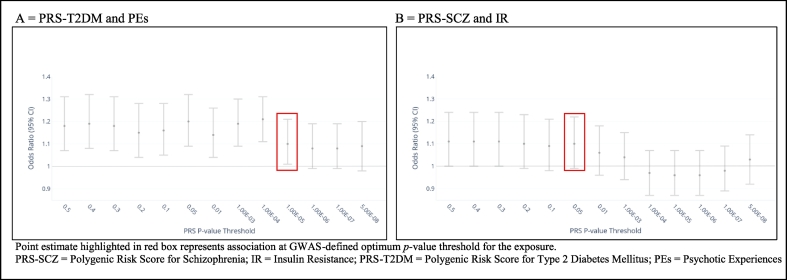
Association between PRS score and outcome at age 18 years at different PRS *P*-value thresholds.

Fig. 2 presents the associations between PRS-T2DM and PEs alongside the associations between PRS-schizophrenia and IR, at different PRS *p*-value thresholds. The point estimates for the PRS-T2DM-PEs associations were >1 for all *p*-value thresholds, though the strength of association weakened at more stringent *p*-value thresholds. A similar pattern was observed for the PRS-schizophrenia-IR association, where the evidence for a positive association attenuated at *p*-value thresholds more stringent than 1.00 × 10^−4^.

### 3.7 Missing data

Fifty-three percent of participants with data on PRS-T2DM had psychotic outcomes data missing, and 71% of participants with PRS-schizophrenia had IR outcome data missing (Supplementary Fig. 1). Compared with the analytic sample, the missing sample had higher mean PRS-schizophrenia but lower PRS-T2DM scores (Supplementary Table 4). Male sex, lower social class and higher BMI predicted missing data for psychotic outcomes, and non-white ethnicity was associated with having missing data for IR (Supplementary Table 5).

## 4 Discussion

### 4.1 Main findings and comparisons with the literature

Using prospective birth cohort data, we report that genetic predisposition for T2DM is associated with psychotic outcomes at age 18 in a linear fashion. The PRS-T2DM findings were consistent using two genetic scores; one with and one without a SNP at the *FTO* locus, which is understood to be related to BMI (Frayling et al., 2007). Additionally, there was evidence for a dose-response pattern in the association between PRS-T2DM and psychotic outcomes; the effect size was strongest for psychotic disorder, which is a more clinically relevant outcome than PEs. We also report some evidence, albeit slightly weaker, for an association between genetic predisposition for schizophrenia and IR at age 18. However, the sample of participants with missing data had higher mean PRS-schizophrenia scores than included participants, thus missing data may help to at least partly explain the weaker evidence. Nonetheless, our findings provide some evidence that the comorbidity between T2DM and schizophrenia arises partly due to shared genetic factors.

The point estimates across various *p*-value thresholds for T2DM and schizophrenia were similar in both combinations of genotype-phenotype analysis, though in both cases at more stringent *p*-value thresholds, the evidence of association weakened. This weakening effect is consistent with a previous study examining the association between PRS-schizophrenia and adolescent psychopathology (Jones et al., 2016), which also reported that PRS-schizophrenia was associated with attrition. Therefore, type II statistical error may be one explanation for the weaker associations between PRS-schizophrenia and IR.

Our results are in line with one previous study in a relatively large sample, which found that people with comorbid schizophrenia and T2DM have a higher genetic predisposition to both disorders compared to controls (Hackinger et al., 2018), and another recent report of an association between PRS for schizophrenia and IR in a clinical sample of people with schizophrenia (Tomasik et al., 2019). Another study found evidence for a genetic overlap between schizophrenia and both triglycerides and HDL (Andreassen et al., 2013), which are cardiometabolic indices known to be tightly linked with an insulin resistance phenotype (Laws and Reaven, 1992), alongside other cardiometabolic factors including systolic blood pressure, BMI and waist: hip ratio. One previous study however found no evidence for an association between PRS-T2DM and schizophrenia (Padmanabhan et al., 2016), though the latter study featured a much smaller sample size than in our study and may therefore have been underpowered to detect a difference. Another study using LD-score regression (Bulik-Sullivan et al., 2015) found limited evidence for a genetic correlation between schizophrenia and T2DM, though the latter study was based on older and less-powered GWAS for both disorders. However, the same study did find some evidence for genetic correlation between schizophrenia and BMI, and, another recent study provides some evidence for shared genetic loci between BMI and mental disorders including schizophrenia (Bahrami et al., 2020). In future, genetic studies may seek to examine the association between PRS scores for other cardiometabolic traits in their association with schizophrenia and other mental disorders.

It is also possible that genetic-risk for T2DM or schizophrenia may increase the risk of both disorders via pleiotropic mechanisms. This may help to explain the differences in our results compared with genetic correlation analyses (Bulik-Sullivan et al., 2015). For example, it is possible that genetic-risk for schizophrenia may predispose to adverse experiences in childhood, which could in t urn influence inflammation (Slopen et al., 2013). We found some evidence for the association of childhood CRP levels with both PRS-T2DM and PRS-schizophrenia. However, we did not find an association with IL-6. This is perhaps unexpected since IL-6 stimulates the production of CRP (Calabro et al., 2003), and is associated with both psychotic outcomes (Khandaker et al., 2014) and IR (Kim et al., 2009). However, it is also possible that genetic predisposition for T2DM or schizophrenia influences CRP via mechanisms other than IL-6. CRP has been shown to play an active role in hepatic insulin resistance, at least partly through impairment in insulin signalling, independent of IL-6 (Xi et al., 2011). Interestingly, CRP has shown to be protective of schizophrenia in MR studies (Hartwig et al., 2017), however, the GWAS studies included in previous MR research measured phenotypic markers in adults. We used CRP measured in childhood, which may be reflective of a distinct biological environment.

We report some evidence that genetic predisposition for T2DM may influence risk of psychosis in early-adulthood by increasing inflammation in childhood, but the magnitude of this mediating effect was small, suggesting that other mechanisms are likely to be involved. On the other hand, we found no evidence that childhood IL-6/CRP mediated the association between genetic predisposition for schizophrenia and IR. The mediating effect of inflammation for the outcome of PEs is consistent with previous research reporting an association between genetic risk for schizophrenia and immune-related disorders (Stringer et al., 2014; Tylee et al., 2018). However, due to the relatively small number of participants with psychotic disorder in our sample and associated lack of power, we were unable to consider testing psychotic disorder in mediation analyses. Future longitudinal research conducted on larger samples of participants may seek to perform a mediation analysis of CRP between PRS-T2DM and more clinically relevant psychotic outcomes.

Other mediators for PRS-T2DM and psychotic outcomes may include non-immune mechanisms such as pleotropic genes affecting distinct biological pathways relevant for each condition. For example, a study examining the genetic overlap between T2DM and schizophrenia highlighted, among others, *PROX1* as a potentially pleiotropic locus (Hackinger et al., 2018). *PROX1* acts both as a transcriptional activator and repressor. It has been implicated in murine beta-cell development as well as in neurogenesis in humans (Holzmann et al., 2015). Due to the relatively small number of participants with psychotic disorder in our sample and associated lack of power, we were unable to consider testing psychotic disorder in mediation analyses. Future longitudinal research conducted on larger samples of participants may seek to perform a mediation analysis of CRP between PRS-T2DM and more clinically relevant psychotic outcomes.

### 4.2 Strengths and limitations

In this study, we have examined the influence of genetic predispositions for T2DM and schizophrenia on, respectively, psychosis-risk and T2DM-risk using a prospective birth cohort. We provide some evidence that a genetic basis may explain at least part of the variance of the commonly observed comorbidity between the two phenotypes. In addition, we have used childhood inflammatory marker data to test potential mediating effects of inflammation for these associations. Since our exposures were genetic risk, the potential for confounding by environmental and lifestyle factors is limited. However, it is well known that certain antipsychotic medications can have adverse effects on glycaemic indices (Leucht et al., 2013). At present, ALSPAC does not have treatment record linkage and we were thus unable to adjust for antipsychotic treatment. This may have impacted our results for the analyses examining PRS-schizophrenia and IR. We were able to control for potential confounding effects of sex, BMI, social class and for inflammatory disease. Regarding ethnicity, participants of non-European genetic ancestry were removed at the stage of genotyping analysis. We also adjusted our regression analyses for ethnicity, since ethnicity is significantly associated with T2DM-risk (Oldroyd et al., 2005). We further adjusted for PCs (Price et al., 2006) in our PRS analyses, to further reduce the risk of population stratification bias. By including PRS for schizophrenia in our analyses, we help to address a common limitation of research conducted on PEs, that they may not adequately capture schizophrenia liability (Jones et al., 2016); the results of both sets of analyses were consistent. A key limitation is missing data. Over half of the risk set with data on PRS had outcome data missing at follow-up. The missing sample had a higher mean score for PRS-schizophrenia but a lower mean score for PRS-T2DM. Thus, our analyses may underestimate the true association between genetic predisposition for schizophrenia and IR, whilst the opposite might be the case for the association between PRS-T2DM and psychotic outcomes. Furthermore, whilst PEs and psychotic disorder have been shown to reflect an increased risk for psychotic disorders (Sullivan et al., 2020; Zammit et al., 2013), and PEs lie on a continuum with clinical psychosis in the general population (van Os et al., 2009), our data do not allow us to determine whether people meet criteria for specific psychotic disorders as classified in DSM or ICD. The transition from PEs to clinical psychosis is low (Kaymaz et al., 2012), PEs are also associated with other psychiatric phenotypes such as depressive and anxiety disorders (Kelleher et al., 2012), and previous research has found no evidence of an association between PRS-schizophrenia and PEs (Jones et al., 2016). Additionally, since our psychotic outcomes were measured prior to the peak age of onset of clinical psychosis (Eranti et al., 2013), some participants may not have yet developed psychotic symptoms or disorder. This point also applies to our sample of participants meeting the criteria for IR at age 18, since age 18 may be relatively early for the phenotype to become detectable. This may be a further explanation for the weaker evidence for an association between PRS-schizophrenia and IR at age 18. Whilst we attempted to address these limitations by reversing the genotype and phenotype to more accurately capture schizophrenia/T2DM liability, replication of our methods in an adequately powered clinical (and likely older) sample of people with clinically diagnosed psychotic disorders such as schizophrenia, is necessary. Finally, one-off measurements of inflammatory markers in childhood may not reflect lifelong levels of inflammation. However, measurement error, if non-differential, introduces a bias towards the null, so our results may underestimate the true association between PRS-T2DM and IL-6 and CRP.

Future research may complement our work by employing genomic advances which test a greater proportion of genomic information than solely PRS scores, the latter of which are highly dependent on the power of GWAS studies. Such methods might include colocalization analysis (Giambartolomei et al., 2014) or locus-level genetic correlation analysis (Shi et al., 2017). Such research may build on our own since research conducted using PRS scores may be susceptible to type II error due to the phenomenon of ‘missing heritability’, which is the difference between the known heritability of a trait compared with the currently identified risk-increasing variants (Manolio et al., 2009). It is likely that at least some of the heritability of schizophrenia (Woo et al., 2017) as well as cardiometabolic disorders (Xia et al., 2016) lies in a number of low-frequency, low-effect-size variants which are therefore difficult to detect with current GWAS methods.

### 4.3 Implications

Our work provides some evidence that, limitations notwithstanding, a summation of minor genetic variation representing lifetime risk for T2DM or schizophrenia at conception, may contribute a portion of the variance of the comorbidity of these disorders in adulthood. Furthermore, we report that genetic predisposition for T2DM may increase risk of PEs by influencing physiologic changes, such as low-grade inflammation, detectable as early as childhood. It is well known that some commonly prescribed antipsychotics can cause or worsen cardiometabolic indices (Leucht et al., 2013), even after a relatively short length of exposure (Neilsen et al., 2010). Therefore, clinicians who look after people with schizophrenia should ascribe detailed attention to the malleable risk factors for cardiometabolic disorders, such as with the promotion of a healthy lifestyle (Teasdale et al., 2019; Ward et al., 2017), and with careful selection and monitoring of antipsychotic medications. This may help to reduce the excess cardiometabolic illness related morbidity and mortality in people with schizophrenia. In future, similar research may seek to examine the associations between PRS for T2DM and other mental disorders including T2DM and bipolar disorder, both of which are known to have higher rates of cardiometabolic disorders than the general population (Martin et al., 2016). Such research may also help to test the specificity of the findings in this study.

## Declaration of competing interest

The authors report no competing interests.

## Appendix A Supplementary data

Supplementary material Image 1
