# Supplementary material for: Common mechanisms for type 2 diabetes and psychosis: Findings from a prospective birth cohort
Source: Schizophr Res. 2020 Sep;223:227–35. doi: 10.1016/j.schres.2020.08.006 (PMC7758839; doi:10.1016/j.schres.2020.08.006)
Supplement: Supplementary file 1 — Supplementary material [file mmc1.docx]

**Online Supplementary Material**

**Perry BI *et al.* Common Mechanisms for Type 2 Diabetes and Psychosis: Findings from a Prospective Birth Cohort**

**Supplementary Methods**

**Supplementary Figure 1: Flowchart of included participants**

PRS-T2DM and PRS Schizophrenia
n=7,977

## n with data on exposure and outcome variables

## n with data on exposure, outcome and confounders

## n with data on exposure

Psychosis outcomes
n=3,768

IR outcome
n=2,344

53% missing data for psychosis outcomes; 71% missing data for IR outcome

Psychosis outcomes
n=3,070

IR outcome
n=1,970

62% missing data for psychosis outcomes; 75% missing data for IR outcome

**Laboratory Methods**

**Genetic data**

Genotype data were acquired by ALSPAC using the Illumina HumanHap550 quad genome-wide single-nucleotide polymorphism (SNP) genotyping platform from 8,812 participants. Individuals were excluded from further analysis by ALSPAC based on gender mismatches, minimal or excessive heterozygosity, disproportionate levels of individual missingness (>3%), evidence of cryptic relatedness (>10% of alleles identical by descent) and being of non-European ancestry (assessed by multidimensional scaling analysis including HapMap 2 individuals). SNPs with a minor allele frequency (MAF) of < 1%, Impute2 information quality metric of < 0.8, a call rate of < 95% or evidence for violations of Hardy-Weinberg equilibrium (p value < 5 x 10-7) were removed. Imputation of the target data was performed using Impute V2.2.2 against the 1000 genomes reference panel (Phase 1, Version 3; all polymorphic SNPs excluding singletons), using 2186 reference haplotypes (including non-Europeans), by ALSPAC. Following quality control assessment and imputation and restricting to 1 young person per family, genetic data was available for 7,977 ALSPAC individuals.

**Measurement of IL-6 and CRP at Age 9 Years**

Blood samples were frozen at −80°C. The measurements were assayed in 2008 after a median of 7.5 years in storage. There was no evidence of freeze-thaw cycles during storage period. IL-6 was measured by ELISA (R&D systems, Abingdon, UK), and CRP was measured by automated particle-enhanced immunoturbidimetric assay (Roche UK, Welwyn Garden City, UK). All assay coefficients of variation were <5%. The minimum detection limit for IL-6 was 0.1pg/mL. This represents the lowest measureable analytic level that can be distinguished from zero. Those below this limit were assigned a value of zero (0.4% of the sample) and were also included in our analysis. The minimum detection limit for CRP was 0.03mg/L. Twenty nine participants (0.6% of the sample) were below this limit, and were assigned values of 0.01 (n=16) and 0.02 (n=13); they were also included in the analysis. 32 subjects had CRP levels >10mg/L and were excluded from analysis due to the risk of acute inflammatory state such as infection, which may have confounded results.

**Supplementary Table 1: Frequency of PEs at Age 18 in the Month Preceding Assessment in the ALSPAC Cohort**

| **Frequency of PEs** | **Number of Participants** | **% with Definite PEs** |
| --- | --- | --- |
| None | 123 | 54.7 |
| Occasionally (1-3 times) | 52 | 23.1 |
| Weekly (4 or more times) | 21 | 9.3 |
| Most days | 7 | 12.9 |

**Supplementary Table 2: Period of Time Since Onset of Psychotic Experiences (PEs) at Age 18 in the ALSPAC Cohort**

| **Period of Time Since Onset of PEs** | **Number of Participants** | **% with Suspected/Definite PEs** |
| --- | --- | --- |
| <3 Months | 29 | 6.6 |
| >3 Months to <1 Year | 115 | 26.1 |
| >1 Year to <5 Years | 158 | 35.8 |
| >5 Years | 139 | 31.5 |

**Supplementary Results**

**Supplementary Table 3: Association between PRS-T2DM (without *FTO* associated SNP) and Psychosis-Risk at Age 18 Years**

| **Risk Factor/outcome** | **No. (%) with Outcome** | **OR (95% C.I.)** | | ***p*-value** | **Corrected *p*-value^c^** |
| --- | --- | --- | --- | --- | --- |
|  |  | **Unadjusted^a^** | **Adjusted for sex, ethnicity, social class and BMI^b^** |  |  |
| **Definite PEs** | | | | |  |
| PRS-T2DM without *FTO* | 183 (5.1) | 1.15 (0.99-1.34) | 1.21 (1.02-1.46) | 0.025 | 0.051 |
| **Psychotic Disorder** | | | | |  |
| PRS-T2DM without *FTO* | 38 (1.0) | 1.42 (1.01-1.96) | 1.50 (1.04-2.03) | 0.016 | 0.048* |

^a^Unadjusted analysis adjusted for 10 principal components only

^b^ Samples for adjusted analysis included 3,070 participants for psychotic outcomes and 1,970 participants for insulin resistance outcome

^c^*p-*value corrected from adjusted analysis using Holm-Bonferroni method

*evidence surpasses Holm-Bonferroni threshold

**Supplementary Table 4: Mean PRS-T2DM and PRS-Schizophrenia in the analytic and missing samples compared using separate variance T-Test**

| **Outcome** | ***n*** | **Mean Z-transformed PRS Score** | **Test Statistic*, p*-value** |
| --- | --- | --- | --- |
| **PRS-T2DM with Missing Psychosis-Risk Data** | | | |
| Analytic sample | 3,768 | 0.033 |  |
| Missing sample | 4,209 | -0.252 | t=3.2, p=0.002 |
| **PRS-Schizophrenia with Missing IR Data** | | | |
| Analytic sample | 2,344 | -0.083 |  |
| Missing sample | 5,633 | 0.334 | t=-4.7, p<0.001 |

**Supplementary Table 5: Predictors of Missing Outcome Data at Age 18 Years for Participants with Data on PRS (*n*=7,977)**

| **Predictor/Outcome** | ***n* (%) with data^1^** | **OR (95% C.I.)** | ***p*-value** |
| --- | --- | --- | --- |
| **Psychosis outcomes** | | | |
| Sex^2^ | 7,870 (99) | 1.69 (1.55-1.84) | <0.001 |
| Ethnicity^3^ | 7,876 (89) | 0.62 (0.11-3.40) | 0.583 |
| Social Class^4^ | 7,060 (89) | 1.11 (1.08-1.13) | <0.001 |
| BMI | 5,062 (63) | 1.05 (1.02-1.07) | 0.001 |
| **IR** | | | |
| Sex^2^ | 7,870 (99) | 1.00 (0.92-1.03) | 0.203 |
| Ethnicity^3^ | 7,876 (89) | 0.51 (0.42-0.60) | <0.001 |
| Social Class^4^ | 7,060 (89) | 1.01 (0.98-1.04) | 0.827 |
| BMI | 5,062 (63) | 1.06 (0.91-1.17) | 0.547 |

^1^*n* with predictor from risk set of all participants with data on PRS (n=8,812)
^2^Female sex is reference
^3^White-British is reference
^4^Social Class I is reference
